# Supplementary material for: A Multi-Experiment Investigation of the Effects Stance Width on the Biomechanics of the Barbell Squat
Source: Sports (Basel). 2022 Sep 14;10(9):136. doi: 10.3390/sports10090136 (PMC9503729; doi:10.3390/sports10090136)
Supplement: Supplementary file 1 [file sports-10-00136-s001.zip › sports-1897359-supplementary.pdf]

Table S1: Kinetic and temporal parameters (mean ± SD) from experiment 1 as a function of each stance-width group.

| | NARROW | | MID | | WIDE | |
|---|---|---|---|---|---|---|
| | Mean | *SD* | Mean | *SD* | Mean | *SD* |
| Peak power (W/kg) | 13.48 | 4.55 | 12.38 | 3.15 | 12.90 | 3.05 |
| Peak bar velocity (m/s) | 0.94 | 0.17 | 0.97 | 0.19 | 1.00 | 0.18 |
| Peak bar acceleration ($m/s^2$) | 4.82 | 1.93 | 4.60 | 1.66 | 4.54 | 1.92 |
| Total squat time (s) | 2.32 | 0.46 | 2.33 | 0.32 | 2.31 | 0.42 |
| Ascent duration (s) | 1.16 | 0.21 | 1.14 | 0.18 | 1.12 | 0.20 |
| Ascent % | 50.46 | 3.68 | 48.89 | 3.61 | 48.97 | 4.84 |
| Peak vertical GRF (N/kg) | 11.97 | 1.19 | 11.67 | 1.16 | 11.81 | 1.17 |
| Vertical RFD (N/kg/s) | 62.42 | 25.24 | 75.81 | 30.58 | 67.01 | 27.48 |
| Vertical GRF ascent impulse (N/kg·s) | 10.74 | 2.51 | 10.57 | 2.02 | 10.45 | 2.81 |
| Vertical GRF descent impulse(N/kg·s) | 11.09 | 3.66 | 11.32 | 2.50 | 11.19 | 3.48 |
| Ankle energy (%) | 9.86 | 3.27 | 8.02 | 2.91 | 7.55 | 2.93 |
| Anterior knee displacement (%) | 49.67 | 9.08 | 45.20 | 10.94 | 45.19 | 9.84 |
| Lateral knee displacement (%) | 22.55 | 10.01 | 26.49 | 6.07 | 24.60 | 7.48 |
| Squat depth (m) | 0.50 | 0.09 | 0.46 | 0.08 | 0.47 | 0.09 |

Table S2: Muscle forces (mean ± SD) from experiment 1 as a function of each stance-width group.

| | NARROW | | MID | | WIDE | |
|---|---|---|---|---|---|---|
| | Mean | *SD* | Mean | *SD* | Mean | *SD* |
| Peak quadriceps force (N/kg) | 74.52 | 21.50 | 75.00 | 17.35 | 74.31 | 17.40 |
| Quadriceps RFD (N/kg/s) | 359.22 | 224.96 | 349.84 | 152.51 | 367.98 | 206.16 |
| Quadriceps ascent impulse (N/kg·s) | 44.53 | 11.38 | 47.16 | 10.81 | 41.83 | 12.27 |
| Quadriceps descent impulse (N/kg·s) | 49.56 | 17.56 | 52.33 | 14.19 | 50.09 | 16.67 |
| Quadriceps force at mid lift (N/kg) | 65.02 | 23.89 | 68.17 | 19.24 | 63.06 | 20.20 |
| Peak gluteus force (N/kg) | 21.16 | 7.54 | 25.04 | 9.86 | 26.65 | 13.69 |
| Gluteus RFD (N/kg/s) | 88.76 | 50.10 | 106.04 | 69.90 | 144.30 | 161.61 |
| Gluteus ascent impulse (N/kg·s) | 9.20 | 3.09 | 10.53 | 3.78 | 11.20 | 3.94 |
| Gluteus force at mid lift (N/kg) | 20.42 | 7.20 | 23.75 | 9.20 | 25.47 | 13.30 |
| Hamstring RFD (N/kg/s) | 159.78 | 137.43 | 195.84 | 146.18 | 249.27 | 203.09 |
| Peak gastrocnemius force (N/kg) | 7.58 | 2.15 | 7.05 | 1.91 | 6.60 | 1.84 |
| Gastrocnemius RFD (N/kg/s) | 26.59 | 9.07 | 28.08 | 9.10 | 26.10 | 10.66 |
| Gastrocnemius descent impulse (N/kg·s) | 4.77 | 2.24 | 4.38 | 1.26 | 4.23 | 1.72 |
| Peak soleus force (N/kg) | 16.18 | 4.59 | 15.05 | 4.08 | 14.09 | 3.92 |
| Soleus RFD (N/kg/s) | 56.62 | 18.94 | 59.59 | 18.70 | 55.79 | 22.84 |
| Soleus descent impulse (N/kg·s) | 10.13 | 4.82 | 9.17 | 2.80 | 8.99 | 3.71 |

Table S3: Three-dimensional kinematics (mean ± SD) as a function of each stance-width group.

| | **NARROW** | | **MID** | | **WIDE** | |
|---|---|---|---|---|---|---|
| | Mean | *SD* | Mean | *SD* | Mean | *SD* |
| Trunk flexion at mid lift (°) | 30.03 | 6.24 | 29.12 | 8.38 | 30.17 | 10.40 |
| Trunk ROM (°) | 28.77 | 5.51 | 26.61 | 5.32 | 26.23 | 7.54 |
| Hip flexion at mid lift (°) | 100.86 | 25.90 | 88.55 | 21.53 | 99.56 | 27.40 |
| Hip internal rotation at mid lift (°) | 9.65 | 12.41 | 4.59 | 11.49 | 7.60 | 12.23 |
| Hip flexion ROM (°) | 90.52 | 21.18 | 75.72 | 20.51 | 84.81 | 23.59 |
| Hip abduction ROM (°) | 17.38 | 6.42 | 21.63 | 4.10 | 17.47 | 9.05 |
| Hip internal rotation ROM (°) | 23.51 | 11.98 | 27.16 | 11.52 | 27.71 | 11.74 |
| Knee flexion at mid lift (°) | 123.35 | 10.55 | 119.63 | 13.82 | 117.87 | 13.07 |
| Knee adduction at mid lift (°) | 1.76 | 8.42 | -3.41 | 7.36 | 0.03 | 7.62 |
| Knee internal rotation at mid lift (°) | 12.77 | 12.88 | 12.81 | 8.75 | 9.42 | 13.46 |
| Knee flexion ROM (°) | 116.72 | 12.68 | 111.38 | 14.05 | 108.67 | 12.71 |
| Knee adduction ROM (°) | 7.95 | 5.74 | 7.19 | 6.66 | 5.11 | 4.72 |
| Knee internal rotation ROM (°) | 17.26 | 13.01 | 12.48 | 9.58 | 14.69 | 8.23 |
| Ankle dorsiflexion at mid lift (°) | 24.49 | 6.14 | 24.30 | 6.48 | 22.88 | 4.50 |
| Ankle dorsiflexion ROM (°) | 26.34 | 4.95 | 24.93 | 5.66 | 24.04 | 4.86 |
| Ankle eversion ROM (°) | 8.14 | 3.72 | 9.81 | 5.22 | 6.97 | 5.60 |
| Ankle internal rotation ROM (°) | 5.52 | 3.15 | 4.54 | 2.96 | 3.75 | 2.60 |

Table S4: Kinetic and temporal parameters (mean ± SD) from experiment 2 as a function of each stance-width condition.

| | **NARROW** | | **MID** | | **WIDE** | |
|---|---|---|---|---|---|---|
| | Mean | *SD* | Mean | *SD* | Mean | *SD* |
| Peak bar acceleration ($m/s^2$) | 4.76 | 1.02 | 4.92 | 1.00 | 4.67 | 1.13 |
| Ascent % | 49.94 | 4.41 | 50.56 | 4.84 | 50.76 | 5.08 |
| Vertical RFD (N/kg/s) | 52.16 | 7.38 | 51.35 | 10.53 | 57.50 | 15.38 |
| Vertical GRF ascent impulse (N/kg·s) | 8.55 | 1.65 | 8.77 | 1.86 | 9.10 | 1.80 |
| Ankle energy (%) | 8.39 | 1.82 | 8.94 | 2.17 | 7.52 | 2.11 |
| Squat depth (m) | 0.50 | 0.02 | 0.50 | 0.03 | 0.49 | 0.03 |

Table S5: Muscle forces (mean ± SD) from experiment 2 as a function of each stance-width condition.

| | **NARROW** | | **MID** | | **WIDE** | |
|---|---|---|---|---|---|---|
| | Mean | *SD* | Mean | *SD* | Mean | *SD* |
| Quadriceps RFD (N/kg/s) | 339.33 | 55.74 | 313.39 | 47.07 | 302.44 | 38.64 |
| Quadriceps ascent impulse (N/kg·s) | 41.91 | 6.97 | 40.91 | 7.21 | 42.28 | 6.54 |
| Quadriceps descent impulse (N/kg·s) | 45.22 | 6.92 | 43.16 | 6.16 | 44.28 | 6.81 |
| Quadriceps force at mid lift (N/kg) | 64.38 | 9.06 | 61.71 | 10.37 | 55.68 | 8.58 |
| Gluteus RFD (N/kg/s) | 104.83 | 43.30 | 103.22 | 34.04 | 124.53 | 61.18 |
| Gluteus descent impulse (N/kg·s) | 14.03 | 6.30 | 14.70 | 6.66 | 18.00 | 11.72 |
| Hamstring RFD (N/kg/s) | 211.81 | 70.07 | 211.87 | 58.03 | 246.53 | 97.32 |
| Gastrocnemius RFD (N/kg/s) | 23.32 | 5.05 | 22.36 | 4.80 | 26.40 | 7.91 |
| Gastrocnemius ascent impulse (N/kg·s) | 4.14 | 0.95 | 4.64 | 1.48 | 4.31 | 1.45 |
| Gastrocnemius descent impulse (N/kg·s) | 3.63 | 1.00 | 3.61 | 1.26 | 3.60 | 1.07 |
| Soleus RFD (N/kg/s) | 49.79 | 10.78 | 47.73 | 10.26 | 56.36 | 16.89 |
| Soleus ascent impulse (N/kg·s) | 8.84 | 2.04 | 9.92 | 3.15 | 9.20 | 3.09 |
| Soleus descent impulse (N/kg·s) | 7.76 | 2.13 | 7.70 | 2.69 | 7.69 | 2.27 |

Table S6: Three-dimensional kinematics (mean ± SD) from experiment 2 as a function of each stance-width condition.

| | **NARROW** | | **MID** | | **WIDE** | |
|---|---|---|---|---|---|---|
| | Mean | *SD* | Mean | *SD* | Mean | *SD* |
| Trunk flexion at mid lift (°) | 30.70 | 5.85 | 32.17 | 6.49 | 33.30 | 6.86 |
| Trunk ROM (°) | 21.53 | 2.95 | 22.11 | 2.84 | 23.74 | 3.74 |
| Hip flexion at mid lift (°) | 95.56 | 13.20 | 95.52 | 14.38 | 97.30 | 15.05 |
| Hip flexion ROM (°) | 73.24 | 16.70 | 72.20 | 17.42 | 71.85 | 18.87 |
| Knee adduction at mid lift (°) | 2.80 | 11.03 | -0.18 | 10.37 | -3.32 | 12.79 |
| Knee internal rotation at mid lift (°) | -4.68 | 12.13 | -7.90 | 11.62 | -10.31 | 11.93 |
| Knee adduction ROM (°) | 8.79 | 5.18 | 7.41 | 6.01 | 8.07 | 8.54 |
| Knee internal rotation ROM (°) | 13.69 | 8.96 | 12.21 | 7.10 | 11.77 | 6.68 |
| Ankle eversion ROM (°) | 4.34 | 2.50 | 4.96 | 1.82 | 3.74 | 2.22 |
| Ankle internal rotation ROM (°) | 3.32 | 3.92 | 2.89 | 3.04 | 3.87 | 2.38 |
